# The role of above-ground competition and nitrogen *vs*. phosphorus enrichment in seedling survival of common European plant species of semi-natural grasslands

**DOI:** 10.1371/journal.pone.0174380

**Published:** 2017-03-23

**Authors:** Tobias Ceulemans, Eva Hulsmans, Sigi Berwaers, Kasper Van Acker, Olivier Honnay

**Affiliations:** 1 Plant Conservation and Population Biology, University of Leuven, Leuven, Belgium; 2 Plant and Vegetation Ecology, University of Antwerp, Antwerp, Belgium; Helmholtz Centre for Environmental Research - UFZ, GERMANY

## Abstract

Anthropogenic activities have severely altered fluxes of nitrogen and phosphorus in ecosystems worldwide. In grasslands, subsequent negative effects are commonly attributed to competitive exclusion of plant species following increased above-ground biomass production. However, some studies have shown that this does not fully account for nutrient enrichment effects, questioning whether lowering competition by reducing grassland productivity through mowing or herbivory can mitigate the environmental impact of nutrient pollution. Furthermore, few studies so far discriminate between nitrogen and phosphorus pollution. We performed a full factorial experiment in greenhouse mesocosms combining nitrogen and phosphorus addition with two clipping regimes designed to relax above-ground competition. Next, we studied the survival and growth of seedlings of eight common European grassland species and found that five out of eight species showed higher survival under the clipping regime with the lowest above-ground competition. Phosphorus addition negatively affected seven plant species and nitrogen addition negatively affected four plant species. Importantly, the negative effects of nutrient addition and higher above-ground competition were independent of each other for all but one species. Our results suggest that at any given level of soil nutrients, relaxation of above-ground competition allows for higher seedling survival in grasslands. At the same time, even at low levels of above-ground competition, nutrient enrichment negatively affects survival as compared to nutrient-poor conditions. Therefore, although maintaining low above-ground competition appears essential for species’ recruitment, for instance through mowing or herbivory, these management efforts are likely to be insufficient and we conclude that environmental policies aimed to reduce both excess nitrogen and particularly phosphorus inputs are also necessary.

## Introduction

Anthropogenic activities have severely altered nutrient fluxes through excess rates of fertilizer application following agricultural intensification and elevated levels of atmospheric deposition, causing an estimated ten- to fiftyfold increase of nitrogen and phosphorus inputs in natural and semi-natural ecosystems worldwide [1–3]. Negative environmental effects of nutrient enrichment are commonly attributed to eutrophication, *i*.*e*. the increased biomass production of dominant plant species following the release from nutrient limited growth due to low nutrient availability [4, 5]. As this increase leads to less light in the understory, plant seedlings and small-statured and slow-growing plant species in the understory of the vegetation are outcompeted for light, resulting in plant species loss. Indeed, artificially supplying light to the understory of experimental grasslands experiencing increased above-ground competition, effectively prevented competitive exclusion of low-statured plant species and supported successful seedling recruitment [6]. Also, a series of global grassland experiments showed that herbivory offsets plant species loss following nutrient enrichment by increasing ground-level light in otherwise more productive vegetation [7].

The results of these studies suggest that competitive exclusion of plant species following eutrophication may be mitigated by controlling grassland biomass productivity, for instance through mowing or herbivory, thereby increasing ground-level light availability [6, 7]. However, evidence from two other fertilization experiments demonstrated that plant species disappeared, despite sufficient levels of light [8, 9]. Furthermore, contrary to the expectations, a global analysis of the relationship between productivity and species richness revealed no consistent drop in species richness at high levels of productivity [10]. In turn, these results seem to suggest that environmental strategies aimed to relax above-ground competition are insufficient to mitigate species loss through competitive exclusion. In an era of continually increasing nutrient enrichment [11], it is therefore crucial to elucidate the interaction between increased nutrients on the one hand and increased above-ground competition on the other.

Identifying appropriate environmental strategies seems contingent upon identifying the precise role of specific nutrients such as nitrogen and phosphorus. Indeed, most research so far has focused solely on nitrogen enrichment, whereas the effects of phosphorus pollution in terrestrial ecosystems have received far less attention [12]. This focus on nitrogen originally stems from the assumption that terrestrial ecosystems are predominantly nitrogen limited and therefore more prone to deleterious effects of nitrogen addition [4, 13]. Indeed, increased productivity of ecosystems has been associated with nitrogen fertilization and atmospheric nitrogen deposition worldwide [14, 15]. Additionally, and as opposed to phosphorus, nitrogen pollution is more widely distributed because of its higher environmental mobility through surface and groundwater flows and distribution via atmospheric deposition [2, 3]. Phosphorus pollution on the other hand is more local, associated with agricultural intensification and disruption of biogeochemical conditions affecting soil phosphorus sequestration [3, 16]. Nevertheless, irrespective of nitrogen levels, phosphorus enrichment has also been associated with increased biomass production in European grasslands [17] and phosphorus limitation appears to promote the occurrence of endangered plant species [18, 19]. Finally, next to a suite of (micro)nutrients that are also involved in patterns of nutrient limitation, recent evidence shows a prevalence of synergistic nitrogen and phosphorus co-limitation of primary production in terrestrial ecosystems worldwide [20–22]. Therefore, there is an additional need to simultaneously assess and disentangle the effects of nitrogen *vs*. phosphorus pollution.

In this study, we specifically examined seedling survival and growth, as opposed to changes in total species richness studied in most experiments, as plants in the seedling stage are particularly sensitive to above-ground competition [6, 23, 24]. As successful seedling recruitment is also essential to maintain viable populations in the long term, it may act as an early warning of future extinction through negative population growth rates [24]. This eliminates possible experimental artefacts owing to a time-lagged response of adult plant individuals that may be able to survive deteriorated environmental conditions for a longer period [23, 24]. Our approach also allowed us to investigate treatment effects separately for all plant species, as the common focus on total species number in many previous studies may mask important patterns of biodiversity change. For instance, when species losses equal species gains, species richness does not decline but species of conservational interest may have gone extinct in favor of more common species.

Our main research goals were i) to examine to what extent different levels of above-ground competition and soil nutrient enrichment have independent effects on seedling survival and growth; and ii) to examine to what extent these possible effects differ between two of the most common environmentally increasing nutrients, nitrogen and phosphorus. To achieve these goals, we performed a full factorial greenhouse experiment with eight common European grassland plant species in grassland mesocosms with addition of nitrogen or phosphorus, or a combination of both, under two different regimes of above-ground competition.

## Material and methods

The experiment was conducted in a greenhouse in Leuven in 2014 (Belgium). The average temperature in the greenhouse was 18.3°C ± 1.8 SD, relative humidity of 69.0% ± 18.0 SD and 49.7 μmol m^-2^ s^-1^± 57.1 SD of light (all averaged over the natural day-night regime during the entire experiment). We performed a factorial experiment using grassland mesocosms. To construct the experimental mesocosms, we filled polymer containers of 42 cm by 38 cm and 10 cm height with a mixture of one part commercially available coarse Rhine sand and two parts of soil collected in a nutrient-poor grassland near the city of Leuven (Belgium). We treated the mesocosms a single time with four different nutrient solutions representing i) a control treatment, in which mesocosms were supplied with 500 mL of a solution of 13.9 g NaCl per liter of deionized water (C-treatment); ii) a nitrogen treatment, supplied with 500mL of a solution of 28.3 g NH_4_NO_3_+13.9 g NaCl per liter (N-treatment, equivalent of 309.9 kg N ha^-1^); iii) a phosphorus treatment with 500 mL of a solution of 16.4 mL concentrated H_3_PO_4_ per liter, buffered with 13 mL of 50% NaOH (P-treatment); and iv) a nitrogen + phosphorus treatment supplied with 500 mL of a nutrient solution, consisting of 250 mL of a solution of 32.8 mL concentrated H_3_PO_4_ per liter buffered with 13mL of 50% NaOH and 250 mL of a solution of 56.6 g NH_4_NO_3_ per liter (NP-treatment, same amounts of nitrogen and phosphorus as in the separate N- and P-treatments). To assess to what extent the nutrient treatments raised nutrient availability in the soil of the mesocosms, we randomly took three soil samples per mesocosm four weeks after the nutrient application. We quantified soil pH using a glass electrode in a 1:25 soil and deionized water mixture shaken for 30 minutes. Soil phosphorus availability was determined through Olsen-P extraction followed by colorimetric analysis using the molybdenum blue method [25]. Soil nitrogen availability was determined by a 1 M KCl extraction of NH_4_^+^ and NO_3_^-^ followed by a colorimetric analysis using a segmented flow analyzer ([25], Skalar, Breda, The Netherlands).

To simulate grassland conditions, we sowed the mesocosms one week after fertilization treatments with 15 g of commercially available seed of *Festuca rubra* L. (Cruydthoeck, The Netherlands), a common grass in European grasslands (S1 Table). We allowed six weeks of growth prior to the start of the experiment, with a single clipping 1 cm above ground level after four weeks. To impose the two different levels of above-ground competition with resident vegetation (*F*. *rubra*), we clipped and removed the grass biomass of half of the mesocosms at 4 cm above ground level (short-clipped treatment) and the other half at 10 cm (long-clipped treatment). We did not include unclipped treatments to avoid confounding the effects of the fertilization treatments and treatments of above ground competition via possible effects of nutrient addition on the growth of the grass matrix. All treatments were replicated 5 times to give a total of 40 experimental grassland mesocosms (4 nutrient treatments x 2 clipping treatments x 5 replicates). The mesocosms were randomly placed in the greenhouse in five blocks consisting of the four nutrient and two clipping treatments. Throughout the experiment, the grass vegetation was kept at the chosen height by consistent clipping, while carefully avoiding the seedlings which were marked with a toothpick. The mesocosms were supplied with equal amounts of deionized water three times per week. We measured light levels (W m^-2^) at ground level in the vegetation after six and eight weeks across all mesocosms during daytime light.

Next, we obtained seeds of *Achillea millefolium* L., *Briza media* L., *Campanula rotundifolia* L., *Hypochaeris radicata* L., *Leucanthemum vulgare* Lam., *Nardus stricta* L., *Rumex acetosa* L. and *Succisa pratensis* Moench. from a commercial grower (Cruydthoeck, The Netherlands). We chose these species because they frequently occur as (co-)dominant species in European semi-natural grasslands across a wide array of different soil types (lowland hay meadows, fen meadows, *Nardus* grasslands, calcareous grasslands; S1 Table). The selection also consists of both dicotyls and monocotyls as well as of clonal and non-clonal species (S1 Table). The seeds were allowed to germinate on vermiculite watered with deionized water in an incubator (16 h at 20°C and 8h at 12°C with 16 h of light). Two weeks after germination, we planted 5 seedlings of each species (except for 8 seedlings of *B*. *media*) in each mesocosm using a uniform grid spacing each seedling 4 cm apart. In the first week after planting, seedlings that died were replaced. Next, seedlings were allowed 16 weeks of growth before calculating seedling survival, expressed as the percentage of surviving seedlings. Seedlings were harvested and above-ground biomass of seedlings was determined after drying at 80°C for 2 days. Next, seedling growth rate was calculated, expressed as the final above-ground biomass divided by the number of days growing (mg day^-1^). This expression does not take the seedling biomass prior to planting into account, as removing the radicle of the seedlings to measure initial above-ground biomass weight would be destructive. This introduced an error, as the biomass at the start of the experiment is not taken into account. However, we measured the total biomass of 20 seedlings per species prior to planting as a control (root and aboveground biomass), and the total biomass of these seedlings prior to planting was consistently less than 1% of the final above-ground biomass weight (all seedlings weighed less than 0.01 mg). This indicates that only a small error is made in the expression of seedling growth rate.

To test for the effects of nitrogen and phosphorus addition on seedling survival and seedling growth rate under the two different clipping regimes, we carried out full factorial Generalized or General Linear Models in SPSS. Seedling survival (Generalized) and seedling growth rate (General) were used as dependent variables. Nitrogen and phosphorus addition, clipping height and the interaction factors between these three treatments were used as independent, explanatory variables (SPSS v20.0, Chicago, USA). In the model of seedling survival, we used a binomial distribution with 5 trials (8 trials for B. media). For seedling growth rate we used a normal distribution. Statistical assumptions were validated prior to modeling. Unfortunately, due to seedling mortality, less than three replicate plants were left in the phosphorus addition or nitrogen and phosphorus addition treatments for the seedling growth rates of *C*. *rotundifolia*, *H*. *radicata*, *N*. *stricta* and *R*. *acetosa* (S2 Table). This meant we could not reliably test for the effect of nitrogen and phosphorus addition, clipping height and their interaction factors, so we excluded these species from quantitative modeling. However, we included these results in the figure depicting growth rates for qualitative interpretation.

## Results

After planting, only twelve seedlings across different treatments died in the first week of the experiment and had to be replaced by new seedlings (3 *B*. *media*, 4 *H*. *radicata* and 5 *S*. *pratensis*). There was a significant difference in mean light availability at ground level in the vegetation of the mesocosms in the short-clipped treatment (71.5±16.8 μmol m^-2^ s^-1^) as opposed to the long-clipped treatment (35.7±7.8 μmol m^-2^ s^-1^). There were no significant differences in light availability among the different nutrient treatments (S1 Fig). The soil mixtures of the mesocosms contained 19.35±1.30 mg N kg^-1^ and 29.79±2.57 mg P kg^-1^ in the control treatment, 44.24±3.23 mg N kg^-1^ and 28.28±1.51 mg P kg^-1^ in the N-treatment, 18.56±1.05 mg N kg^-1^ and 66.44±4.27 mg P kg^-1^ in the P-treatment and 42.05±2.44 mg N kg^-1^ and 63.23±3.36 mg P kg^-1^ in the NP-treatment (S2 and S3 Figs). These levels are comparable to a gradient of soil nutrient levels in European grasslands (S4 and S5 Figs). Soil pH did not differ significantly between treatments (S6 Fig).

**Fig 1:**
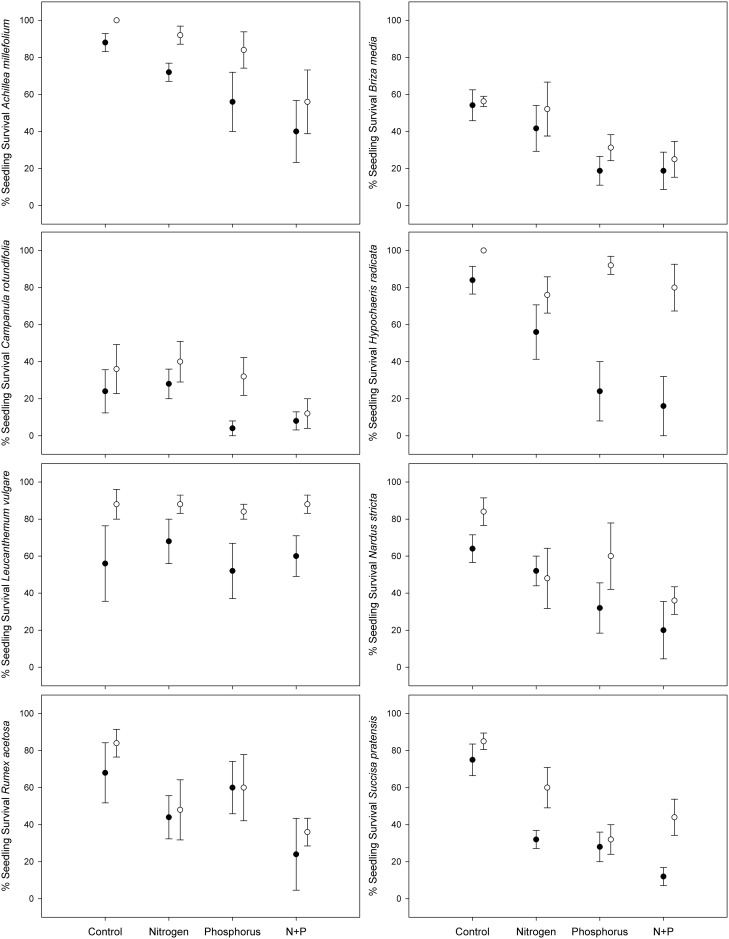
Seedling survival rate of eight common European grassland species across different nutrient addition treatments in experimental grassland mesocosms. Seedling survival is expressed as the % of surviving seedlings after 16 weeks of growth. Open circles represent short-clipped treatment, closed circles represent long-clipped treatment. Data are mean ± standard error of 5 trial individuals in 5 replicate mesocosms per treatment (8 trial individuals for *B*. *media*).

Except for *R*. *acetosa*, *N*. *stricta* and *B*. *media*, seedling survival was significantly higher in the treatment with grass clipped at 4 cm (lower above-ground competition) as opposed to the treatment with grass clipped at 10 cm (higher above-ground competition). In addition, we found that seedling survival of four out of eight species was significantly lower in the nitrogen treatments as opposed to the control treatment (*A*. *millefolium*, *H*. *radicata*, *R*. *acetosa* and *S*. *pratensis*; Table 1, Fig 1). On the other hand, seedling survival of seven out of eight species was significantly lower in the phosphorus treatments as opposed to the control treatment (*A*. *millefolium*, *B*. *media*, *N*. *stricta*, *C*. *rotundifolia*, *R*. *acetosa*, *S*. *pratensis* and *H*. *radicata*; Table 1, Fig 1). Simultaneous phosphorus and nitrogen addition significantly negatively affected seedling survival of *S*. *pratensis* even further (N*P interaction, Table 1, Fig 1). The seedling survival of only one species, *L*. *vulgare*, was unaffected by the different nutrient treatments (Table 1, Fig 1). The response of seedling survival to nutrient addition of *H*. *radicata* was dependent on the clipping treatment (clipping*P interaction), showing that the negative effect of phosphorus addition on the seedling survival of this species is mitigated by relaxation of the above-ground competition (Fig 1).

**Table 1:** Results of the statistical models. The dependent variables were seedling survival, modeled as the survival success out of 5 trials with a binomial distribution in Generalized Linear Model, and seedling growth rate (mg day^-1^) modeled with a normal distribution in a General Linear Model. We could not analyze growth rate of *C*. *rotundifolia*, *H*. *radicata*, *N*. *stricta* and *R*. *acetosa* as there were too few surviving seedlings in at least one treatment. Nitrogen addition, phosphorus addition and clipping treatment and their interaction factors were the explanatory variables.

**Treatment**	*Achillea millefolium*		*Briza media*		*Campanula rotundifolia*	*Hypochaeris radicata*	
	χ^2^_seedling survival_	R^2^_growth rate_	χ^2^_seedling survival_	R^2^_growth rate_	χ^2^_seedling survival_	χ^2^_seedling survival_	
	44.5***	0.1	36.6***	0.11	18.8**	82.8***	
Intercept	31.5***	124.7***	26.4***	78.5***	46.7***	17.8***	
Clipping	10.0**	8.3**	3.0	7.9**	6.1*	27.5***	
Nitrogen	8.2**	0.4	1.3	1.1	0.2	9.7**	
Phosphorus	17.0***	0.6	30.4***	0.7	9.4**	11.5**	
N * P	0.5	0.1	0.1	0.2	1.1	2.3	
Clipping * N	1.3	0.1	0.4	0.6	1.1	1.4	
Clipping * P	1.3	0.2	0.0	0.5	0.6	4.2*	
	*Leucanthemum vulgare*		*Nardus stricta*		*Rumex acetosa*	*Succisa pratensis*	
	χ^2^_seedling survival_	R^2^_growth rate_	χ^2^_seedling survival_		χ^2^_seedling survival_	χ^2^_seedling survival_	R^2^_growth rate_
	22.2**	0.46***	35.7***		25.6***	47.4***	0.62***
Intercept	39.4***	1121.1***	0.3		0.9	1.2	821.2***
Clipping	18.0***	32.2***	3.3		1.5	7.0**	40.5***
Nitrogen	0.7	3.6	1.5		18.0***	7.4**	5.9*
Phosphorus	0.4	0.4	27.8***		5.6*	22.8***	2.4
N * P	0.0	0.6	0.1		0.0	4.3*	5.2*
Clipping * N	0.1	0.0	0.3		0.0	2.8	4.6*
Clipping * P	0.0	0.9	0.7		0.1	0.1	0.4

*0.01<*P*<0.05,

**0.001<*P*<0.01,

***P<0.001.

**Fig 2:**
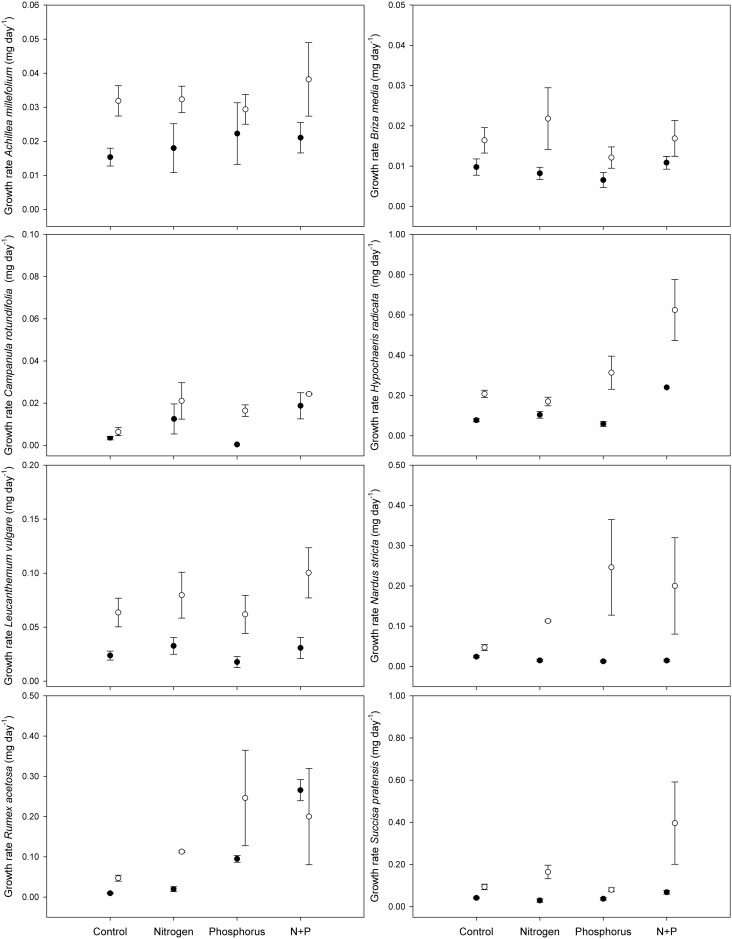
Seedling growth rate of eight common European grassland species across different nutrient addition treatments in experimental grassland mesocosms. Seedling growth rate is expressed as mg of biomass increment day^-1^ over 16 weeks of growth. Open circles represent short-clipped treatment, closed circles represent long-clipped treatment. Data are mean ± standard error of 5 replicate individuals per mesocosm.

Seedling growth rate of all species was higher in the treatments with lower above-ground competition (Table 1, Fig 2). Furthermore, seedling growth rate of *S*. *pratensis* was significantly increased by nitrogen addition (Table 1, Fig 2) and *S*. *pratensis* also showed a significant increase in seedling growth rate following simultaneous phosphorus and nitrogen addition (N*P interaction, Table 1, Fig 2). Qualitatively, seedling growth rate of *N*. *stricta*, and *R*. *acetosa* seemed to be increased by phosphorus addition and by nitrogen addition for *C*. *rotundifolia* and *R*. *acetosa* (Fig 2). Similar to its seedling survival, seedling growth rate of *L*. *vulgare* remained unaffected by the different nutrient treatments (Table 1, Fig 2). *S*. *pratensis* showed an increase in seedling growth rate following nitrogen addition and simultaneous nitrogen and phosphorus addition. However, this increase was dependent on the clipping regime, with a higher seedling growth rate under lower levels of above-ground competition (Table 1; Fig 2).

## Discussion

The most common hypothesis to predict environmental consequences of nutrient enrichment predicts competitive exclusion owing to an increase in above-ground biomass production (e.g. [6, 23]). Our results support this hypothesis by showing reduced seedling survival in the long-clipped treatment, designed to simulate higher above-ground competition, for all but the two grass species in the experiment. Furthermore, seedling growth rate was consistently higher in the short-clipped treatments. However, under the assumption that higher above-ground competition is uniquely responsible, the short-clipped treatment, designed to relax above-ground competition, should cancel out the negative impact of adding nutrients. This was not the case as all but one species (*L*. *vulgare*) showed a decline in seedling survival after nitrogen and/or phosphorus fertilization across both clipping treatments. The negative effects of nutrient addition on seedling survival of only one species was (partly) alleviated by lower levels of above-ground competition in the short-clipped treatments (*H*. *radicata*). However, lower above-ground competition did not fully alleviate its reduced seedling survival, indicating that unique negative effects of nutrient enrichment persist. These results suggest that higher levels of above-ground competition will negatively influence population recruitment, irrespective of nutrient enrichment. In other words, at any given level of soil nutrient availability, lower vegetation productivity should also allow for higher seedling survival. However, our results also indicate that fertilization negatively impacts species’ recruitment irrespective of the extant above-ground competition as all investigated species but *H*. *radicata* showed reduced seedling survival, despite lower levels of competition. Furthermore, only *S*. *pratensis* showed increased seedling growth rates under low competition, indicating nitrogen and phosphorus co-limited growth, which ultimately may alleviate the negative effects of nutrient enrichment on seedling survival.

The consistently high mortality of seedlings in the long-clipped treatment may be attributed to light deprivation as we found lower levels of available light in this treatment as opposed to the short-clipped treatment [6]. Lower light levels in the long-clipped treatment may also account for the consistently lower growth rates, as low light availability limits levels of photosynthesis controlling growth rate [26]. However, inevitably there are some drawbacks of our approach which complicate attributing the observed higher seedling survival and growth rate in the short-clipped treatment to increased light availability. Firstly, short clipping may also support seedling growth through increased soil temperature. However, differences in soil temperature between the short-clipped treatment and long-clipped treatment were limited in this experiment (18.1±0.5°C *vs*. 17.8±0.8°C at 0.5 cm depth). Clipping may also have caused increased below-ground competition as the clipped grass needs to acquire additional nutrients for the renewal of above-ground biomass [9, 23, 27]. However, if this were the case, we would expect lower growth rates and lower seedling survival in the short-clipped conditions, contrary to our results. Furthermore, evidence from a competition experiment manipulating light conditions with clipping, shade cloths and vegetation tie-backs also suggests that the main effects of clipping could be attributed to altered light conditions [9].

The observed lower seedling survival independent of levels of competition on the other hand, corroborates results of experiments showing unique negative effects of nutrient enrichment via mechanisms other than increased biomass production [9, 27]. Furthermore, in addition to separating effects of soil nutrient enrichment on the one hand and above-ground competition on the other, our results also show that the effect of the type of nutrient pollution may differ between plant species. Generally, it would appear that phosphorus enrichment may affect more plant species through a higher reduction of seedling survival, as opposed to nitrogen enrichment. Whereas only half of the tested species were negatively affected by nitrogen addition, increased phosphorus negatively affected the seedling survival of seven out of eight species. In addition, all four species impacted by nitrogen were also negatively impacted by phosphorus, with nearly a double reduction in seedling survival compared to the control treatment under phosphorus enrichment as opposed to nitrogen enrichment for *A*. *millefolium*, *H*. *radicata*, and *S*. *pratensis* (Fig 1). Furthermore, an additive effect of nitrogen and phosphorus addition was only apparent for the seedling survival of *S*. *pratensis*, suggesting that phosphorus enrichment overrides the negative effects of nitrogen enrichment for the other two species. These results are in line with observational evidence relating increased soil phosphorus availability to a lower occurrence of *A*. *millefolium*, *B*. *media*, *H*. *radicata* and *S*. *pratensis*, and of *A*. *millefolium*, *H*. *radicata*, *R*. *acetosa* and *S*. *pratensis* along a gradient of increased atmospheric nitrogen deposition and soil nitrogen availability in semi-natural grasslands across Europe [26–28].

The possible mechanisms underlying detrimental effects of nitrogen and phosphorus pollution include, amongst others, increased soil toxicity, increased susceptibility to pathogens and altered symbiotic relationships [4, 18]. For instance, nitrogen enrichment can cause soil acidification and subsequently cause mineral nutrient deficiencies in plants (such as K^+^, Mg^2+^ and Ca^2+^) or increase availability of toxic metals such as Al^3+^ [29]. Nitrogen enrichment may also lead to toxic accumulation of ammonium, which has been shown to cause reduced seedling survival of *S*. *pratensis*, and of similar plant species characteristic of nutrient poor grasslands and heathlands [30]. Phosphorus enrichment on the other hand has been related to the loss of mycorrhizally dependent plant species, possibly due to altered cost-benefit relationships of mycorrhizally-mediated phosphorus uptake [31]. It has also been shown that phosphorus enrichment may cause the loss of suitable symbiotic partners [32, 33]. Finally, phosphorus enrichment may cause a competitive disadvantage for species specialized in access to recalcitrant chemical forms of soil phosphorus compared to species that show the best access to readily-available ortho-phosphate, undermining plant species coexistence through phosphorus resource partitioning [34].

Although the precise mechanisms were not investigated here, our results show that at any given level of above-ground competition, soil nutrient enrichment will also negatively affect seedling survival compared to nutrient poor conditions. The extent and speed of the impact of both drivers are likely contingent upon the specific species composition of the plant communities and the specific environmental context. For instance, communities dominated by long-lived plant species may not show an immediate decrease in plant species richness, as species extinction owing to impaired seedling recruitment may only become apparent after a few generations [23–24]. However, plant communities may quickly lose species if both adult and seedling plants are susceptible to nutrient enrichment.

In conclusion, although increased above-ground competition following eutrophication appears to present a problem for successful seedling survival, management efforts focusing on lowering community biomass alone are likely to be insufficient to maximize population recruitment. As both drivers seemed to exert a largely independent influence, our results point to both increased above-ground competition, possibly through reduced light availability on the one hand, and unique effects of increased soil nutrient availability on the other, as the main drivers of environmental change following nutrient pollution. Therefore, it seems that environmental policies should combine management of above-ground biomass production, such as mowing or herbivory, with low soil nutrient availability by reducing both excess nitrogen and, particularly, phosphorus input in grasslands.

## Supporting information

S1 Fig Mean light availability across different nutrient addition and clipping treatments in the experimental grassland mesocosms. Open circles represent short-clipped treatment, closed circles represent long-clipped treatment. Data are mean ± standard error.
(DOCX)

S2 Fig Soil mineral nitrogen (NH_4_^+^+NO_3_^-^) determined by 1M KCl extraction across different nutrient addition treatments in the experimental grassland mesocosms (Robertson *et al*. 1999). Open circles represent short-clipped treatment, closed circles represent long-clipped treatment. Data are mean ± standard error.
(DOCX)

S3 Fig Soil phosphorus determined by Olsen-extraction across different nutrient addition treatments in the experimental grassland mesocosms (Robertson *et al*. 1999). Open circles represent short-clipped treatment, closed circles represent long-clipped treatment. Data are mean ± standard error.
(DOCX)

S4 Fig Relationship between soil mineral nitrogen (NH_4_^+^+NO_3_^-^) determined by 1M KCl extraction and plant species number as observed in 132 grasslands surveyed across Northwestern Europe. Data from Ceulemans *et al*. 2013. Reference lines represent mean nitrogen levels measured in the four different nutrient addition treatments in the experimental grassland mesocosms (see S2 Fig).
(DOCX)

S5 Fig Relationship between soil phosphorus determined by Olsen-extraction and plant species number as observed in 501 grasslands surveyed across Europe. Data from Ceulemans *et al*. 2014. Reference lines represent mean phosphorus levels measured in the four different nutrient addition treatments in the experimental grassland mesocosms (see S3 Fig).
(DOCX)

S6 Fig Soil pH across different nutrient addition treatments in the experimental grassland mesocosms (Robertson *et al*. 1999). Open circles represent short-clipped treatment, closed circles represent long-clipped treatment. Data are mean ± standard error.
(DOCX)

S1 Table Occurrence of the selected plant species in European grasslands. Occurrence is based on data of species composition of 4m^2^ quadrats in 501 European grasslands spread across 10 European countries (data from Ceulemans *et al*. 2014). The species frequently occur as (co-)dominant species in lowland hay meadows (1), fen meadows (2), *Nardus* grassland (3), calcareous grassland (4). They consist of both dicotyls and monocotyls as well as clonal and non-clonal species (Fitter & Peat 1994, The Ecological Flora Database, *J*. *Ecol*., 82, 415–425).
(DOCX)

S2 Table Raw data of seedling survival of eight common European grassland species across different nutrient addition treatments in experimental grassland mesocosms. Seedling survival is expressed as the number of surviving seedlings after 16 weeks of growth of 5 trial individuals (8 trial individuals for *B*. *media*).
(DOCX)

S3 Table Raw data of the seedling growth rate of eight common European grassland species across different nutrient addition treatments in experimental grassland mesocosms. Seedling growth rate is expressed as mg of biomass increment day^-1^ over 16 weeks of growth. Missing data is due to seedling mortality.
(DOCX)
