# Supplementary material for: The role of above-ground competition and nitrogen vs. phosphorus enrichment in seedling survival of common European plant species of semi-natural grasslands
Source: PLoS One. 2017 Mar 23;12(3):e0174380. doi: 10.1371/journal.pone.0174380 (PMC5363941; doi:10.1371/journal.pone.0174380)
Supplement: S2 Fig — Open circles represent short-clipped treatment, closed circles represent long-clipped treatment. Data are mean ± standard error. (DOCX) [file pone.0174380.s002.docx]

**
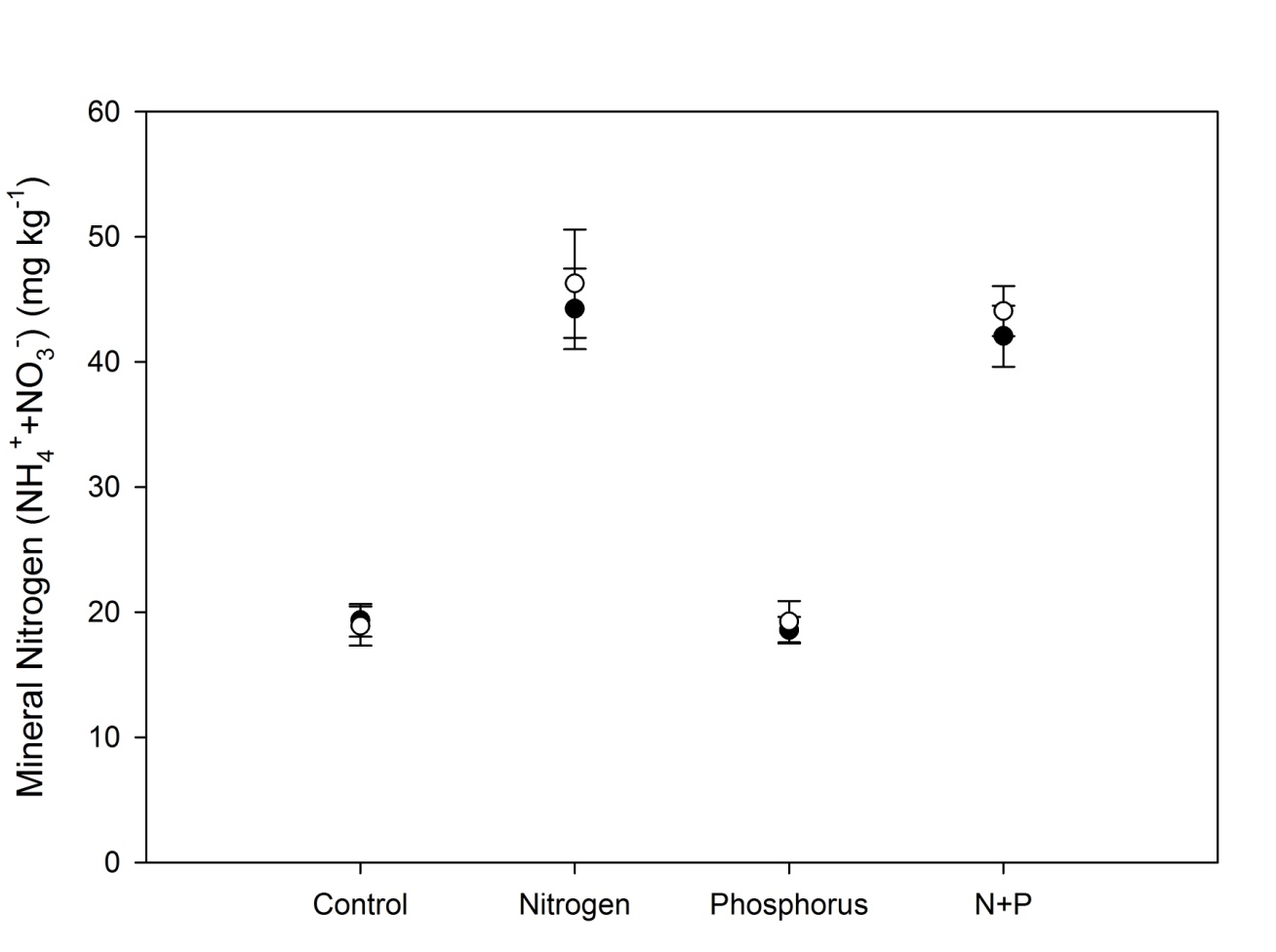
**

**S2 Figure.** Soil mineral nitrogen (NH_4_^+^+NO_3_^-^) determined by 1M KCl extraction across different nutrient addition treatments in the experimental grassland mesocosms (Robertson *et al.* 1999). Open circles represent short clipped treatment, closed circles represent long clipped treatment. Data are mean ± standard error.
