# Supplementary material for: The role of above-ground competition and nitrogen vs. phosphorus enrichment in seedling survival of common European plant species of semi-natural grasslands
Source: PLoS One. 2017 Mar 23;12(3):e0174380. doi: 10.1371/journal.pone.0174380 (PMC5363941; doi:10.1371/journal.pone.0174380)
Supplement: S4 Fig — Data from Ceulemans et al. 2013. Reference lines represent mean nitrogen levels measured in the four different nutrient addition treatments in the experimental grassland mesocosms (see S2 Fig). (DOCX) [file pone.0174380.s004.docx]

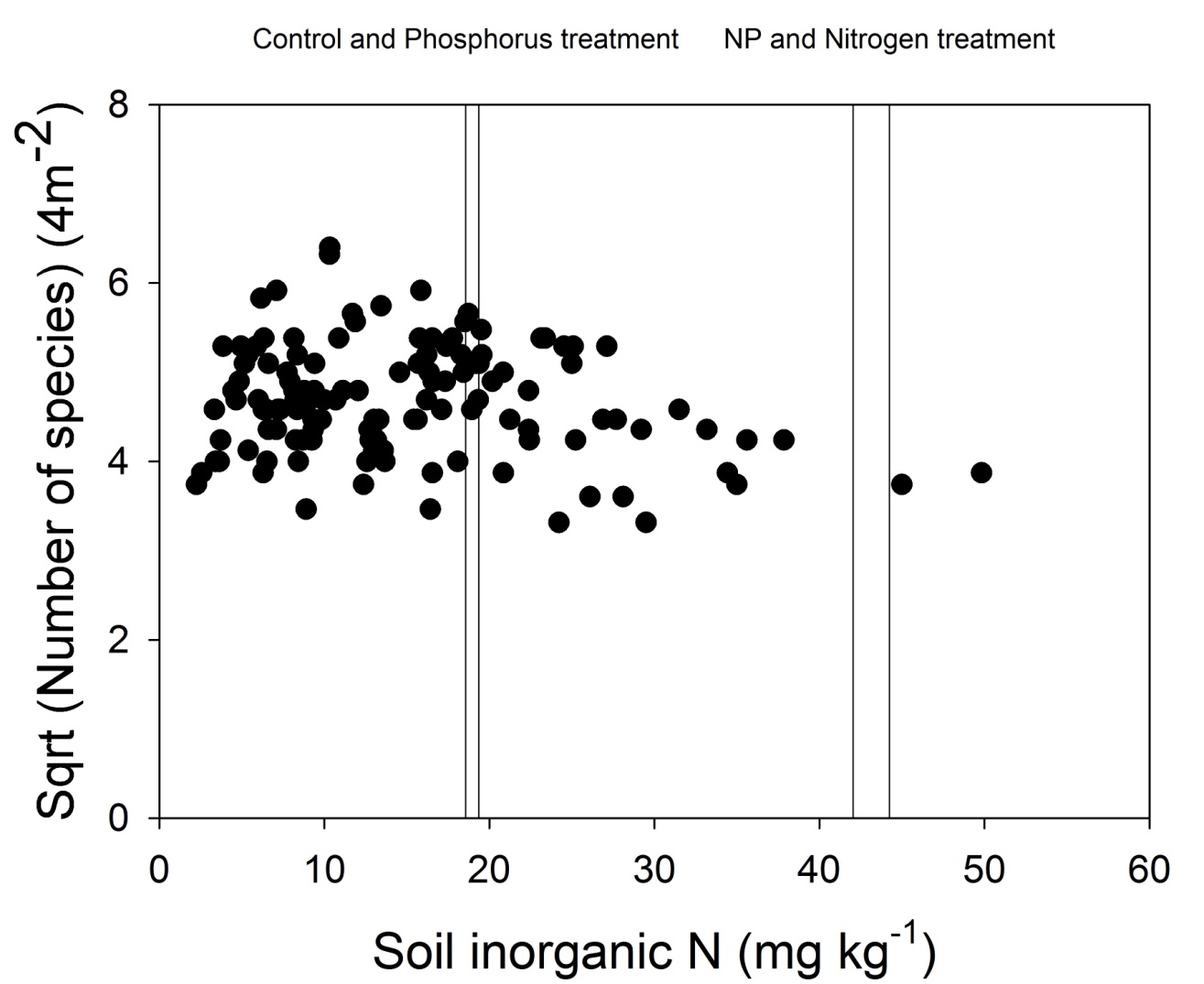


**S4 Figure.** Relationship between soil mineral nitrogen (NH_4_^+^+NO_3_^-^) determined by 1M KCl extraction and plant species number as observed in 132 grasslands surveyed across Northwestern Europe. Data from Ceulemans *et al.* 2013. Reference lines represent mean nitrogen levels measured in the four different nutrient addition treatments in the experimental grassland mesocosms (see Figure S2).
