# Supplementary material for: The role of above-ground competition and nitrogen vs. phosphorus enrichment in seedling survival of common European plant species of semi-natural grasslands
Source: PLoS One. 2017 Mar 23;12(3):e0174380. doi: 10.1371/journal.pone.0174380 (PMC5363941; doi:10.1371/journal.pone.0174380)
Supplement: S5 Fig — Data from Ceulemans et al. 2014. Reference lines represent mean phosphorus levels measured in the four different nutrient addition treatments in the experimental grassland mesocosms (see S3 Fig). (DOCX) [file pone.0174380.s005.docx]

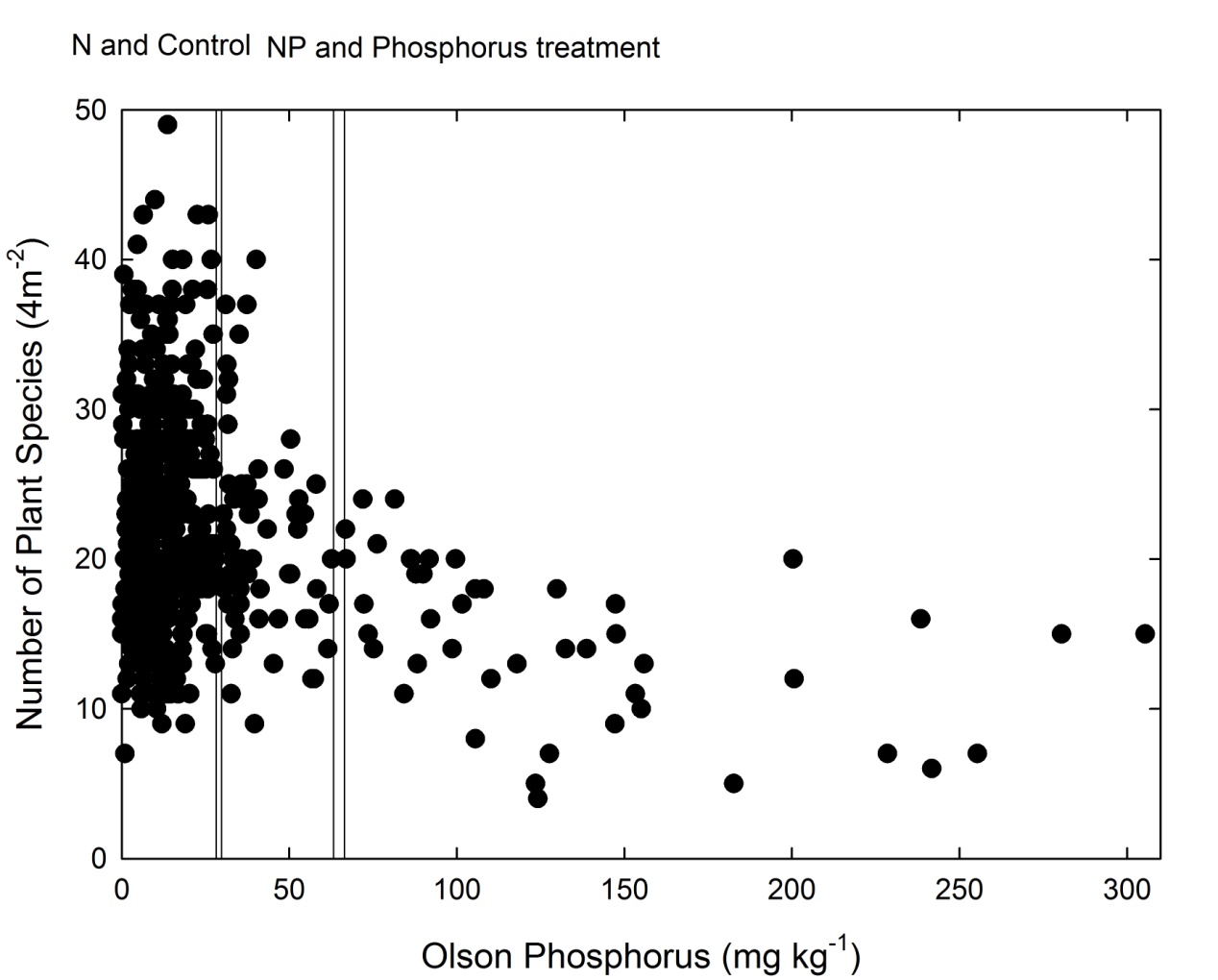


**S5 Figure.** Relationship between soil phosphorus determined by Olson-extraction and plant species number as observed in 501 grasslands surveyed across Europe. Data from Ceulemans *et al.* 2014. Reference lines represent mean phosphorus levels measured in the four different nutrient addition treatments in the experimental grassland mesocosms (see Figure S3).
