# Supplementary material for: The role of above-ground competition and nitrogen vs. phosphorus enrichment in seedling survival of common European plant species of semi-natural grasslands
Source: PLoS One. 2017 Mar 23;12(3):e0174380. doi: 10.1371/journal.pone.0174380 (PMC5363941; doi:10.1371/journal.pone.0174380)
Supplement: S1 Table — Occurrence is based on data of species composition of 4m2 quadrats in 501 European grasslands spread across 10 European countries (data from Ceulemans et al. 2014). The species frequently occur as (co-)dominant species in lowland hay meadows (1), fen meadows (2), Nardus grassland (3), calcareous grassland (4). They consist of both dicotyls and monocotyls as well as clonal and non-clonal species (Fitter & Peat 1994, The Ecological Flora Database, J. Ecol., 82, 415–425). (DOCX) [file pone.0174380.s007.docx]

**Table S1. Occurrence of the selected plant species in European grasslands.** Occurrence is based on data of species composition of 4m^2^ quadrats in 501 European grasslands spread across 10 European countries (data from Ceulemans *et al.* 2014). The species frequently occur as (co-)dominant species in lowland hay meadows (1), fen meadows (2), *Nardus* grassland (3), calcareous grassland (4). They consist of both dicotyls and monocotyls as well as clonal and non-clonal species (Fitter & Peat 1994, The Ecological Flora Database, *J. Ecol.*, 82, 415-425).
