# Supplementary material for: The role of above-ground competition and nitrogen vs. phosphorus enrichment in seedling survival of common European plant species of semi-natural grasslands
Source: PLoS One. 2017 Mar 23;12(3):e0174380. doi: 10.1371/journal.pone.0174380 (PMC5363941; doi:10.1371/journal.pone.0174380)
Supplement: S2 Table — Seedling survival is expressed as the number of surviving seedlings after 16 weeks of growth of 5 trial individuals (8 trial individuals for B. media). (DOCX) [file pone.0174380.s008.docx]

**Table S2. Raw data of seedling survival of eight common European grassland species across different nutrient addition treatments in experimental grassland mesocosms.** Seedling survival is expressed as the number of surviving seedlings after 16 weeks of growth of 5 trial individuals (8 trial individuals for B. media).
