# Supplementary material for: The role of above-ground competition and nitrogen vs. phosphorus enrichment in seedling survival of common European plant species of semi-natural grasslands
Source: PLoS One. 2017 Mar 23;12(3):e0174380. doi: 10.1371/journal.pone.0174380 (PMC5363941; doi:10.1371/journal.pone.0174380)
Supplement: S3 Table — Seedling growth rate is expressed as mg of biomass increment day-1 over 16 weeks of growth. Missing data is due to seedling mortality. (DOCX) [file pone.0174380.s009.docx]

**Table S3. Raw data of the seedling growth rate of eight common European grassland species across different nutrient addition treatments in experimental grassland mesocosm**s. Seedling growth rate is expressed as mg of biomass increment day^-1^ over 16 weeks of growth rounded to 3 decimals. Missing data is due to seedling mortality.
